# Ground Tire Rubber Modified by Elastomers via Low-Temperature Extrusion Process: Physico-Mechanical Properties and Volatile Organic Emission Assessment

**DOI:** 10.3390/polym14030546

**Published:** 2022-01-28

**Authors:** Paulina Wiśniewska, Łukasz Zedler, Mariusz Marć, Marek Klein, Józef Haponiuk, Krzysztof Formela

**Affiliations:** 1 Department of Polymer Technology, Faculty of Chemistry, Gdańsk University of Technology, Gabriela Narutowicza 11/12, 80-233 Gdańsk, Poland; paulina.wisniewska1@pg.edu.pl (P.W.); lukasz.zedler@pg.edu.pl (Ł.Z.); jozef.haponiuk@pg.edu.pl (J.H.); 2 Advanced Materials Center, Gdańsk University of Technology, Gabriela Narutowicza 11/12, 80-233 Gdańsk, Poland; 3 Department of Analytical Chemistry, Faculty of Chemistry, Gdańsk University of Technology, Gabriela Narutowicza 11/12, 80-233 Gdańsk, Poland; mariusz.marc@pg.edu.pl; 4 Institute of Fluid Flow Machinery, Polish Academy of Sciences, Fiszera 14, 80-231 Gdańsk, Poland; marek.klein@imp.gda.pl

**Keywords:** ground tire rubber, extrusion, modification, rubber recycling, physico-mechanical properties, volatile organic compounds

## Abstract

In this paper, low-temperature extrusion of ground tire rubber was performed as a pro-ecological waste tires recycling method. During this process, ground tire rubber was modified with constant content of dicumyl peroxide and a variable amount of elastomer (in the range: 2.5–15 phr). During the studies, three types of elastomers were used: styrene-butadiene rubber, styrene-ethylene/butylene-styrene grafted with maleic anhydride and ethylene-octene copolymer. Energy consumption measurements, curing characteristics, physico-mechanical properties and volatile organic compounds emitted from modified reclaimed GTR were determined. The VOCs emission profile was investigated using a passive sampling technique, miniature emission chambers system and static headspace analysis and subsequently quantitative or qualitative analysis by gas chromatography. The VOCs analysis showed that in the studied conditions the most emitted volatile compounds are dicumyl peroxide decomposition by-products, such as: α-methylstyrene, acetophenone, α-cumyl alcohol, methyl cumyl ether, while the detection level of benzothiazole (devulcanization “marker”) was very low. Moreover, it was found that the mechanical properties of the obtained materials significantly improved with a higher content of styrene-butadiene rubber and styrene-ethylene/butylene-styrene grafted with maleic anhydride while the opposite trend was observed for ethylene-octene copolymer content.

## 1. Introduction

Tires are complex and high-performance composites, which consist of many components such as tread, tread base, tread chimney, cushion, sidewall, bead region, plies, belts, overlay, shoulder wedge, inner liner, gum strips, etc. [1]. Each component has a different function (wear, durability, cushioning, noise and vibration dampening, and traction), and consequently, each has a different composition, including rubbers, vulcanizing agents, accelerators, activators, antiozonants, antioxidants, retarders, plasticizers, and fillers. Depending on the manufacturer, up to fourteen different compounds can be found in a tire, not including the types of steel cord and fabric reinforcement [1]. Such a composition is necessary to obtain a product that meets the high requirements; however, it becomes a major drawback at the end of its life.

Waste tires management and recycling are challenging tasks, which are related to their complex composition and the cross-linked structure. Nevertheless, there are some widely used methods for managing waste tires [2,3,4,5,6,7,8,9,10,11,12,13]. At present, the most popular approach in waste tires utilization is energy recovery or obtaining liquid or gaseous fuel (burning, pyrolysis, gasification, etc.). Other methods lead to the production of materials that directly contact the environment and people (civil engineering, reclaiming/devulcanization). This means that these products must meet the appropriate requirements set for them by manufacturers, customers, and standards, such as the easiest processing method, specific physico-mechanical characteristics, and characterization of the potential risk to the site and product users.

From the point of view of processability and physico-mechanical properties of waste rubber-based products the use of GTR alone, or the introduction of its unmodified form into polymer matrices, leads to a significant deterioration of the properties of the final product [14,15,16,17,18]. This problem can be overcome by suitable GTR modification via extrusion, which seems to be one of the most promising methods for this purpose. Temperature and shear force act on the material during extrusion, allowing not only scission of the cross-links and main chains, which translates to an improvement of flowability, but also oxidizing the surface, resulting in the appearance of hydroxyl groups [19]. New functional groups in the GTR surface might be resulting in improved interactions between GTR and matrix. Moreover, it allows simultaneous modification of the GTR and mixing with a polymer, which improves the components’ compatibility by applying high shear [20,21].

However, most GTR modifications, in the presence of a polymer, by extrusion are carried out at high temperatures (150–270 °C) [22,23,24,25]. To enhance the compatibilization of the system, shear forces must be increased, which can be achieved by lowering process temperature. Recent studies indicate that the use of lower temperatures leads to materials with satisfactory physico-mechanical properties, reduced energy consumption, and a reduction in the generation and emission of VOCs [26,27,28].

It was proven that high-temperature GTR processing favors the generation and emission of toxic gases such as dioxins, furans, carbon dioxide, hydrogen sulfide, and sulfur dioxide into the atmosphere [29,30]. This means that even if the amount of VOCs generated is reduced by using low-temperature extrusion, the resulting material is a potential source account for releasing hazardous substances into the environment.

A study conducted by the Office of Environmental Health Hazard Assessment for the State of California on the potential risks of using waste rubber from car/truck tires in public facilities [31] shows that the use of GTR-based pavements is associated with a risk of releasing to the atmosphere compounds containing fifteen of metals (among them arsenic, lead, and mercury), twenty volatile organic compounds (methyl ethyl ketone, toluene, benzene, polycyclic aromatic hydrocarbons), fourteen semi-volatile (among them benzothiazole, aniline) and particulates in the air resulting from tire wear. Mohajerani et al. [32] published a review paper on the use of waste rubber from the automotive industry, focusing on geotechnical engineering applications. In addition to the economic and utility aspects, they also focused on the environmental impact. Their work indicated that Hg (0.12 mg/L) and Al (1.81 mg/L) were detected in leachate. Moreover, they also showed the presence of benzothiazole in the amount of 0.45–0.54 mg/L.

Janajreh et al. [33] conducted the PAH analysis for a GTR-based tile used for playgrounds detecting twelve compounds in the total concentration of 214 µg/g. The analytical measurements also confirmed the presence of compounds such as phthalates, adipates, antioxidants, and benzothiazole with high concentrations reaching as high as 3 mg/g. Another study on the risk of using GTR-based products conducted by Birkholz et al. [34] assessed the human health hazard, as well as environmental toxicity. Their results indicated that no test meets the criteria for genotoxicity. In terms of environmental hazards, the authors have shown that the toxicity of leachates from GTR before and after aging (three months) differs significantly (59% reduction). In conclusion, the authors highlighted that fresh rubber crumb shows moderate toxic threat to aquatic species while it undergoes quick degradation by natural processes.

The available literature on the topic does not clearly indicate specific trends on the environmental impact of GTR-based products. The conclusions drawn depend on the methodology, material, processing method, or environmental factors. However, this discrepancy points to the need for additional analysis of waste tires processing technology to determine the substances that may be emitted from the product and during the production. This need arises because there are still no standardized methods or testing strategy for GTR-based materials.

Zanetti et al. [35] investigated the gaseous emissions generated during the processing of bituminous mixtures containing recycled rubber and their impact on human health. The authors indicated that sampling and laboratory analyses of gaseous emissions are the key factors during workers’ health risk assessment, showing the necessity for the development of methodology and reference database in this field.

This issue was also recently highlighted by Skoczyńska et al. [36], who analyzed recycled rubbers and their recycling products—mats dedicated for roofing and flooring applications. The authors developed an analytical method based on sonication, solid-phase extraction, and gas chromatography combined with mass spectrometry analysis, which gave the best results for extraction and further analysis of aromatic compounds present in ground rubber. Investigation of commercially available recycled rubber mats showed the level of heterocyclic aromatic compounds, which exceed the EU limits for articles placed on the market for use by the public. Surprisingly, for one product these limits were exceeded even a few hundred times.

In our previous work [28], we demonstrated the possibility of obtaining thermoplastic-modified GTR prepared via low-temperature extrusion. With a relatively small addition of ethylene-vinyl acetate copolymer, we have noted an improvement of tensile properties, which were superior to trans-polyoctenamer (an additive commonly used in waste rubber recycling) modified GTR. An equally important conclusion of this study was the determination of generated TVOCs. We found that the addition of the polymeric additive reduced the amount of emissions twofold. Moreover, our studies about low-temperature reclaiming of GTR showed that VOCs analysis should be done in every step of GTR processing in order to evaluate changes in the structure of processed material and its influence on the environment [37].

This work is a continuation of our investigations about GTR modification and the volatile organic compounds emission profile related to this process. This strategy provides useful information about the impact of GTR modification procedures and prepared materials on the environment and human health. Determination of the VOCs emission profile is crucial for further implementation of novel technologies or polymeric materials at an industrial scale.

In this study, GTR was modified by dicumyl peroxide and three different elastomers: (i) styrene-butadiene rubber; (ii) styrene-ethylene/butylene-styrene rubber grafted with maleic anhydride; and (iii) ethylene-octene copolymer. The modification was carried out via low-temperature extrusion. The effects of relatively low elastomer content (in the range of: 2.5–15 phr) on the processing and performance properties of modified GTR were investigated by measurement of energy consumption, the temperature of GTR after treatment, curing behavior, tensile properties, and equilibrium swelling. Moreover, a comprehensive analysis of volatile organic compounds’ emission profiles was conducted.

## 2. Materials and Methods

### 2.1. Materials

In the study, the following components were used to prepare samples for testing:

- Ground tire rubber (GTR)—obtained from passenger and truck tires, with particle sizes up to 0.6 mm, was received from Grupa Recykl S.A. (Śrem, Poland). GTR composition determined by thermogravimetric analysis showed: rubbers and additives (62.3 wt.%), carbon black (26.9 wt.%), silica and ash content (10.8 wt.%). Two peaks related to the presence of natural rubber and styrene-butadiene rubber were observed on differential thermogravimetry plots, confirming that recycled rubber was prepared from waste tires [37].
- Styrene-butadiene rubber (KER 9001)—is a high styrene resin containing about 83% of styrene bonded in the polymer (SBR), and it is characterized with softening point at 35–40 °C, hardness 65–75 Shore D, and volatile matter maximum of 1 wt.% The rubber was supplied by Synthos Rubbers (Oświęcim, Poland).
- Styrene-ethylene/butylene-styrene grafted with maleic anhydride with tradename TAIPOL SEBS 7126—it is characterized by bond maleic anhydride content 1.2–1.8 wt.%, melt flow index (5 kg at 230 °C) 15–25 g/10 min, and volatile matter maximum 0.5 wt.%. The copolymer was supplied by TSRC Corporation (Kaohsiung, Taiwan).
- Ethylene-octene copolymer (EOC) with tradename Solumer 851L—is characterized with melt flow index (2.16 kg at 190 °C) 1 g/10 min and glass transition temperature at −59 °C. The copolymer was supplied by SK Global Chemical Co., Ltd. (Seoul, Korea).
- Dicumyl peroxide (DCP)—organic peroxide commercially used for the curing of unsaturated polyester resins, natural and synthetic rubbers, as well as polyolefins. It is characterized by a peroxide assay minimum of 98% and an active oxygene assay minimum of 5.8%. The peroxide was supplied by Pergan GmbH (Bocholt, Germany).

**Figure 1:**
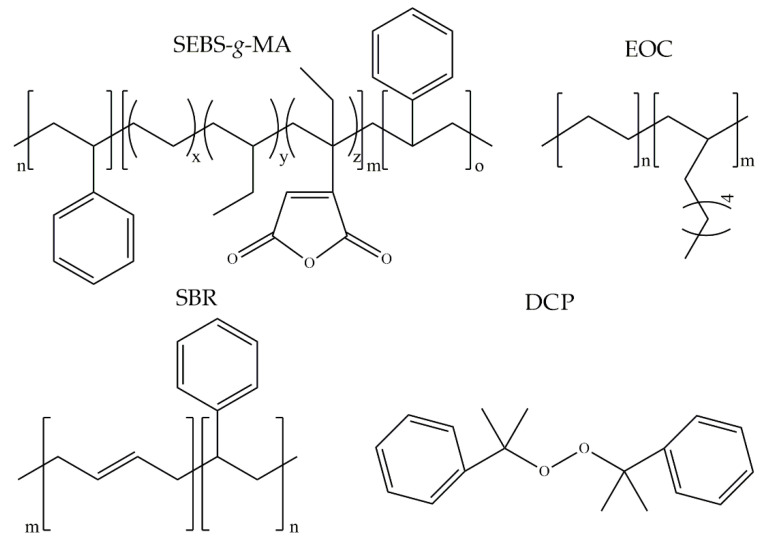
Structural formulas of components used in the study.

The structural formulas of the above components are presented in Figure 1 to better understand the changes that occur during the proposed research.

### 2.2. Sample Preparation

Sample coding, GTR modification and formulation procedure are summarized in Table 1.

### 2.3. Characterization Methods

The energy consumption during reactive extrusion of modified GTR was determined by two methods. The first is based on reading the energy consumption from an electricity meter. The values reported included the energy consumption of all extruder components. Moreover, the specific mechanical energy (SME, expressed in kWh/kg), which determines the energy consumption of the drive motor, was calculated according to Equation (1):

(1) $$SME=\frac{N}{Q}$$

where: *N* is the drive motor power consumption (kW) and *Q* is a throughput (kg/h).

The temperature distribution of modified reclaimed rubber was measured using infrared thermal imaging camera model Testo 872 (Testo SE & Co. KGaA, Lenzkirch, Germany), directly from the die of the extruder.

The vulcanization process was investigated and recorded via Premier RPA Alpha Technologies (Hudson, OH, USA) according to ISO 6502 standard. Further calculations of the cure rate index (CRI) and R_300_ parameter were made in order to determine characteristic values for curing curves. CRI is related to cross-linking rate, while R_300_ parameter indicates the deviation of the cross-linking curve from the plateau. Both parameters were calculated based on equations presented in works [38,39].

The tensile strength and elongation at break were measured in accordance with ISO 37. Tensile tests were carried out on a Zwick Z020 machine (Ulm, Germany) at a 500 mm/min constant speed. Direct extension measurements were conducted using an extensometer with sensor arms. The reported results are an average of five measurements for each sample. Shore hardness type A was assessed using a Zwick 3130 durometer (Ulm, Germany) according to ISO 7619-1.

The density was determined based on the Archimedes method, as explained in ISO 1183. Measurements were carried out at room temperature in a methanol medium, without exception.

The swelling degree of the vulcanized samples (0.2 g) was estimated via a swelling test carried out in toluene at room temperature. The swelling degree was calculated according to Equation (2) as follows:

(2) $$Q=\frac{m_{t}-m_{o}}{m_{o}}\times 100\%$$

where *Q*: swelling degree, %; *m_t_*: a sample mass swollen after time t, g; and *m_o_*: an initial mass of the sample, g.

Sol fraction was calculated by Formula (3) as follows:

(3) $$\mathrm{Sol}\;\mathrm{fraction}=\frac{W_{1}-W_{2}}{W_{1}}\times 100\%\;$$

where *W*_1_*:* mass of the vulcanized sample before swelling, g; and *W*_2_: mass of the vulcanized sample after extraction, g.

According to the following Flory–Rehner Equation (4) [40], cross-link density can be determined by equilibrium swelling in toluene:

(4) $$\nu_{e}=\frac{-\left[\ln(1-V_{r}\right)+V_{r}+\chi V_{r}^{2}]}{\left[V_{1}\left(V_{r}^{1/3}-V_{r}/2\right)\right]}$$

where *ν_e_*: cross-link density, mol/cm^3^; *V_r_*: gel volume in the swollen sample; *V*_1_: solvent molar volume; and *χ*: polymer-solvent interaction parameter.

Additionally, the Kraus correction dedicated for filled compounds [41] was used in order to calculate the actual remaining cross-link density.

The content of elements in uncured and cured modified GTR was determined by the wavelength dispersive X-ray fluorescence spectrometry (WD-XRF) using a 1 KW S8 Tiger spectrometer from Bruker AXS (Karlsruhe, Germany). Samples were analyzed in powder test vessels on Prolene^®^ foil of 4 μm thickness. Measurements were performed in a helium atmosphere.

Volatile organic compounds (VOCs) emitted from reclaimed GTR were determined by static headspace and gas chromatography-mass spectrometry (SHS-GC-MS) techniques. Measurements were performed using a Shimadzu GC2010 PLUS GC-MS (Shimadzu Corporation, Kioto, Japan) equipped with a split/splitless inlet. The GC-MS system was equipped with an AOC5000 Headspace Auto-Sampler. During analysis, the vial was transported by the injection unit from the tray to the agitator; when the sample achieved equilibrium, the headspace sample of 2.5 mL volume was drawn from the vial and injected into the GC injector. The sampled vial was then returned by the injection unit to the tray.

A sampling of VOCs emitted to the gas phase/indoors during reactive extrusion, and RPA measurement was performed using passive sampling technique with Radiello^®^ system (Fondazione Salvatore Maugeri, Padova, Italy). The liberation process of VOCs collected on the Carbograph 4 was performed using a two-stage thermal desorption technique (TD). Liberated analytes were determined with the use of gas chromatography technique equipped with flame ionization detector (GC-FID), as well as GC combined with a mass spectrometer (GC-MS). The TD-GC-FID system (Markes Series 2 Thermal Desorption Systems; UNITY/TD-100; Agilent Technologies 7820A GC System, Santa Clara, CA, USA) was equipped with GC capillary column DB-1. In the case of TD-GC-MS system (Markes Unity v.2, Markes International, Inc., Bridgend, United Kingdom; Agilent Technologies 6890; 5873 Network MSD, Agilent Technologies, Santa Clara, CA, USA), the applied capillary column was HP-1MS. In both TD units, the extraction of analytes from solid sorbent Carbograph 4 was performed in the splitless mode as well as the gas flow rate during the desorption from the microtrap was the same as the carrier gas flow rate through the applied columns of the GC system. Obtained analytical information describes the mass of analytes emitted to the gas phase during the reactive extrusion and adsorbed on Radiello^®^ sorption medium (Carbograph 4). Due to the fact that the employed passive samplers worked in relatively unfavorable conditions, the estimation of the concentration of the determined VOCs in the gas phase was not the subject of the research. More detailed information regarding used stationary equipment and methodology are listed in Supplementary Materials: Tables S1 and S2, as well as presented in works [42,43,44].

In the case of emissions of VOCs from prepared modified GTR samples, the studies were carried out with the use of a miniature emission chambers system µ-CTE™ 250 (Markes’ Micro-Chamber/Thermal Extractor™, Markes International, Inc., Bridgend, UK) [45,46,47]. Analytes adsorbed on a sorption medium Tenax TA were liberated using the above-mentioned TD units under parallel conditions. The separation, identification, and final determination processes were performed using previously described GC-FID and GC-MS systems in analogues working parameters. The only difference was the need to use a split (approx. 1:30) during sample injection in the case of the GC-MS system to avoid overloading the detector and the GC column (small internal diameter and relatively thin film of the stationary phase). Detailed information about the emission studies conditions and equipment parameters are enclosed in the Supplementary Materials: Tables S2 and S3. In addition, information about the full operating parameters and analytical procedures in which µ-CTE™ 250 was used, was enclosed in detail elsewhere [44,47,48,49,50].

Identification and quantification of the main representatives of VOCs emitted from the investigated samples as well as collected by Radiello^®^ passive sampler were carried out on the basis of certified reference material (VOC EPA Mix 2, Supelco, Bellefonte, PA, USA) containing 2000 µg/mL of each of the 13 compounds (including benzene, toluene, styrene, ethylbenzene, and representative of xylenes) in 1 mL of methanol. Identification in the TD-GC-FID system was made on the basis of a comparison of the retention times obtained for the chemicals in samples with the retention time of the analytes for the mentioned certified reference VOCs mixture. The quantification of the VOCs representatives was performed based on the external standard technique. The seven-point calibration solutions in methanol were prepared, containing desired analytes in the range from 2 ng/µL up to 2000 ng/µL. The calibration protocol was carried out according to the procedure and equipment described in detail elsewhere [51,52] and in Supplementary Materials. The limit of detection was assessed based on a signal-to-noise (S/N) ratio and the average value of this parameter was 0.30 ng.

The total amount of volatile organic compounds (TVOCs parameter) in both types of studies was calculated considering the protocol in which the TVOC parameter is the sum of all VOCs, characterized by retention time between n-hexane and n-hexadecane in a case of non-polar or slightly polar GC column stationary phases using FID quantifying as toluene equivalents [53,54].

Identification in the TD-GC-MS system was performed in an analogous manner. For other compounds, the identification was performed using the mass spectra database (NIST 2.0 Mass Spectral Library) included in the mass spectrometer software (The NIST Mass Spectral Search Program for the NIST/EPA/NIH Mass Spectral Library Version 2.0d, build 2 December 2005, copyright by the U.S. Secretary of Commerce on behalf of the United States of America and FairCom Corporation, Sandy, UT, USA) (only relationships with a probability above 90% agreement were considered). As for the aliphatic and aromatic hydrocarbons not included in the applied VOCs reference mixture but identified on the GC-MS system, the chromatograms received on the TD-GC-MS system were compared with chromatograms received with the use of the TD-GC-FID system, and their amounts were assessed based on FID response factors and a determined calibration curve of toluene (present in an above mentioned VOCs reference standard solution) [55].

## 3. Results and Discussion

### 3.1. Temperature and Energy Consumption Measurements

**Figure 2:**
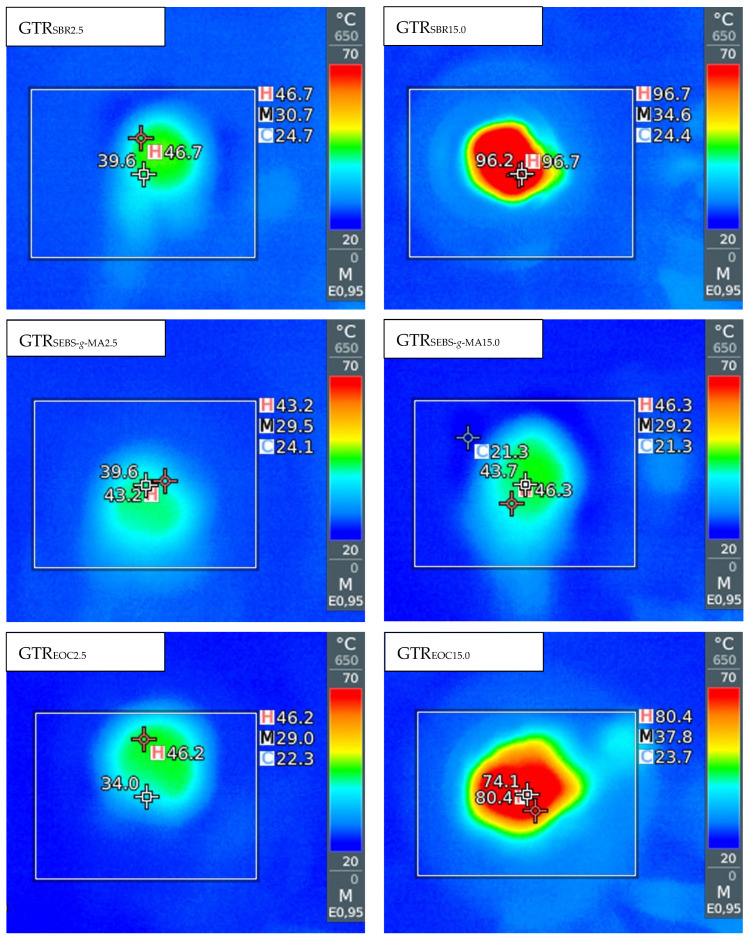
The infrared camera images for selected samples.

The temperature and energy consumption measured during GTR modification are summarized in Table 2. In order to better evaluate the temperature distribution at the extruder die, the infrared camera images are shown in Figure 2.

It was observed that a relatively small amount of modifier (5 phr EOC or 10 phr SBR) used during low temperature devulcanization of GTR strongly affects the temperature of the material after extrusion. The highest temperature at a die was measured for GTR_EOC15.0_ and GTR_SBR15.0_ which achieved even 81 ± 1 °C and 96 ± 1 °C, respectively, while the temperatures in the individual heating zones on the barrel of the extruder were set to 35/60/60/60/60/60/60/25/25/25 °C. This proves the self-heating phenomenon of the modified GTR as a result of increased friction caused by shear forces. However, this effect was rather limited in the case of SEBS-*g*-MA. This can be related to possible reactions between GTR and this type of modifier, which affects the processing behavior of modified GTR as presented in the next subsection. Moreover, it is worth mentioning that the addition of modifiers also affects the appearance of processed materials. In contrast to pure GTR, which after reclaiming process, is in the form of powder [28], the modified GTR takes the shape of solid profiles. It can therefore be said that these compounds act as a binder of the rubber particles.

The energy consumption measurements during processing provide valuable information about process efficiency and determine the possibility of its application at an industrial scale. As presented in Table 1, extruder energy consumption during GTR modification was in the range of 0.407–0.587 kWh/kg. The most power (22–45% of total energy consumption) is related to the drive motor, determined by the SME parameter. It was observed that the highest values of SME were measured for materials characterized by the highest temperature at die. As mentioned, it results from the increased friction between the rubber particles and the modifier matrix, which creates higher torque during extrusion.

### 3.2. Curing Characteristics of Modified GTR

**Figure 3:**
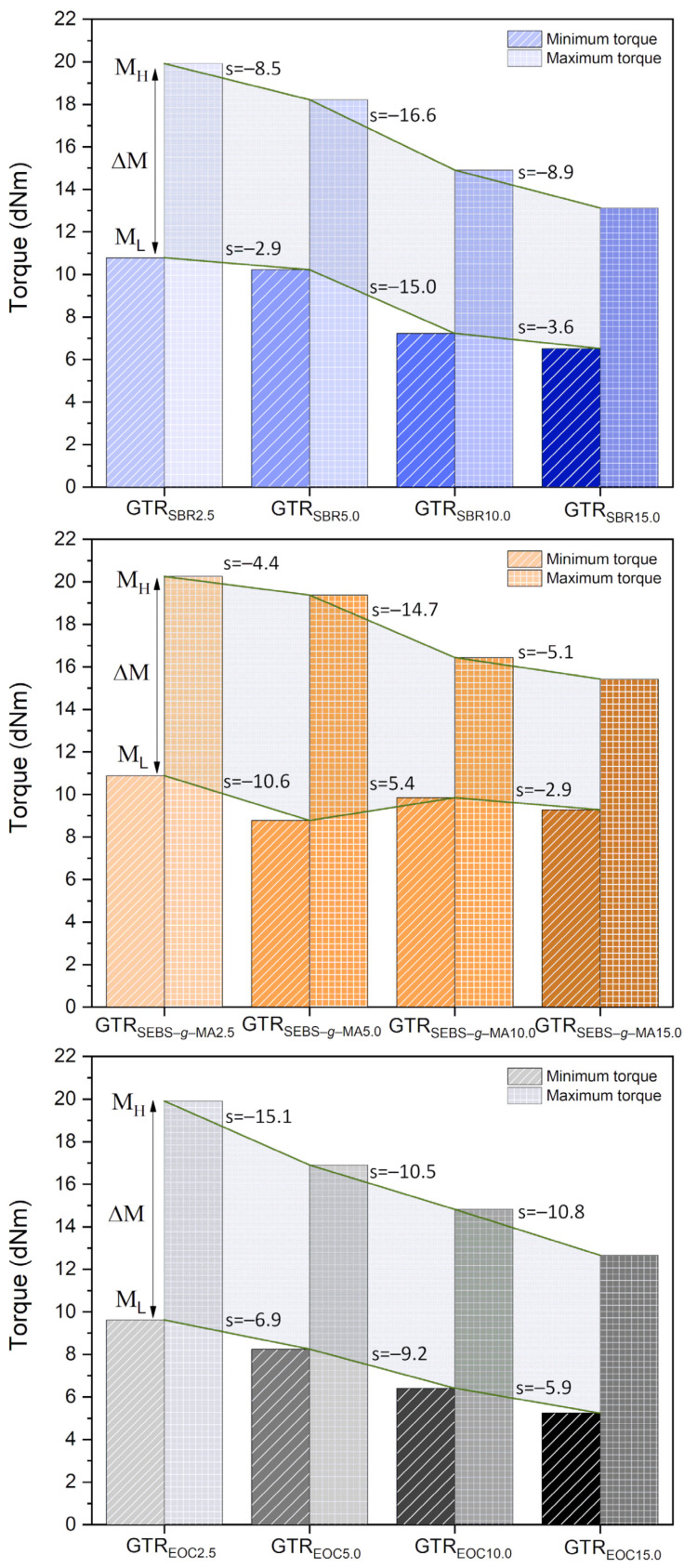
Torque parameter depending on the type and content of the modifier (curing characteristics at 170 °C).

The effect of the type and content of modifiers used on the curing characteristics of GTR is shown in Figure 3 and summarized in Table 3. Minimum torque (M_L_) is a parameter that proves the processing properties of the materials. It was noted that with the increase in modifier content (EOC or SBR), the processing capacity improved, as evidenced by the decrease in the M_L_ parameter from 9.6 dNm to 5.2 dNm (for GTR modified by EOC) and from 10.8 dNm to 6.5 dNm (for GTR modified by SBR). Unlike the materials mentioned above, the processability of the SEBS-*g*-MA modified GTR did not improve significantly with the higher additive content. Comparing the M_L_ values with different types of modifiers used, it can be concluded that the EOC modified GTR had the best processing properties which originate from a simpler structure of the modifier, not containing spatial structures (such as phenyl groups). The opposite observation is made with regard to the stiffness of the materials, which is characterized by the parameter of maximum torque. The highest values of M_H_ were noted consecutively for GTR modified by SEBS-*g*-MA, SBR, EOC. Moreover, in all cases, the increase in modifier content contributed to a reduction in the stiffness of the materials. The same trend is noticeable for torque increment (∆M), which refers to the effectiveness of the cure. Materials with higher modifier content were characterized by a lower extent of cure because mainly modified GTR is involved in the cross-linking process. Regardless of the type and content of the modifier used, the scorch time and optimum cure time were comparable for the investigated samples and were within the range of 0.1–0.6 min and 5.1–6.3 min, respectively. This means that the cross-linking rate was similar, which is confirmed by the constant values of the cure rate index. This is related to using the same cross-linking system for each sample, which determines the curing characteristics of the studied materials. The thermal aging resistance parameter was in the range of 0.2–0.9%, which indicates good thermal stability of modified GTR during curing at 170 °C.

### 3.3. Physico-Mechanical Properties of Modified GTR

**Figure 4:**
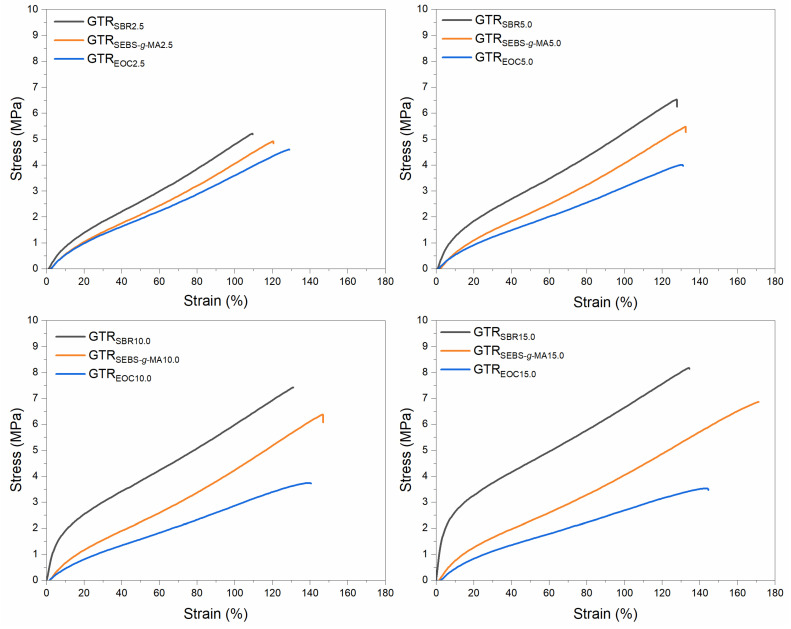
Stress–strain curves determined for modified GTR.

The physico-mechanical properties of modified GTR are summarized in Table 4. For a better comparison of the obtained results, the stress-strain curves are presented in Figure 4. For a higher amount of modifier in GTR, a slight increase in elongation at break was observed. The most significant change was noted for SEBS-*g*-MA modified GTR, from 120 ± 6% to 170 ± 5%. Moreover, it can be seen that the higher content of the SEBS-*g*-MA or SBR modifier, the higher value of tensile strength was determined. The maximum value of this parameter reached 6.9 ± 0.1 MPa (for GTR_SEBS-*g*-MA15.0_) and 8.1 ± 0.3 MPa (for GTR_SBR15.0_). Surprisingly, the presence of EOC in modified GTR contributed to the deterioration of the tensile strength. It decreased proportionally from 4.5 ± 0.1 MPa (GTR_EOC2.5_) to 3.5 ± 0.3 MPa (GTR_EOC15.0_). This may be related to the low stiffness of the material, which also contributed to the slight reduction of hardness. For GTR modified by SEBS-*g*-MA a constant value of hardness (66–67 Shore A) was noted, while for GTR modified by SBR a significant improvement in this parameter (from 71 to 84 Shore A) was observed with the increasing content of the modifier.

The materials’ density was in the range of: 1.100–1.162 g/cm^3^. With the increase in the modifier content in all types of modified GTR samples the density of the materials tended to decrease. It is related to the lower density of EOC, SEBS-*g*-MA, and SBR additive compared to GTR. Another essential property that was investigated is the cross-link density of modified GTR. It can be noticed that the increase in the modifier content contributed to the increase in swelling degree and thus a decrease in cross-link density of the materials. The cross-link density changed significantly from 1.13 ± 0.02 to 0.64 ± 0.02 mol/cm^3^ × 10^−4^ (GTR_EOC_), from 1.25 ± 0.04 to 0.77 ± 0.01 mol/cm^3^ × 10^−4^ (GTR_SEBS-*g*-MA_), and from 1.30 ± 0.07 to 0.92 ± 0.01 mol/cm^3^ × 10^−4^ (GTR_SBR_). The lowest cross-link density was characterized by the EOC modified GTR, which results from better flow in relation to other materials.

Among the produced materials, SBR modified GTR showed the best performance properties. In Table 5, the mechanical properties of these samples were compared with GTR/SBR blends investigated by other research groups. The presented tensile strength values [56,57,58,59] are significantly lower than those determined in this paper, although the content of waste rubber was smaller in the materials tested by other research groups. Only in [60] a higher value of this parameter was achieved but it is believed that it is caused by the presence of polypropylene matrix. By comparing the presented data, a conclusion can be drawn that not only the composition of materials has a significant impact on final properties. Contrary to expectation, the tensile strength of SBR/GTR samples with the ratios 50/50, 40/60, and 20/80 obtained by different research groups was 4.9, 5.0, and 6.0 MPa, respectively. It proves that especially important are processing conditions and in the case of GTR, also the effectiveness of the devulcanization process.

### 3.4. XRF Analysis of Modified GTR

In order to better analyze the composition of modified GTR, X-ray fluorescence spectrometry was applied and the obtained results are shown in Table 6. This method allows for the identification of only heavier elements in studied materials; therefore, the presence of carbon was not detected. The most intensive signals detected from XRF spectroscopy correspond to zinc, silicon, and sulfur. The high content of zinc (about 1%) results from the presence of zinc stearate or zinc oxide in reclaimed rubbers, a commonly used activator for rubber compounding. The presence of silicon (0.40–1.28%) indicates the occurrence of silica, a popular filler used in tires to increase their abrasion resistance. The presence of silica might be also related to impurities present in waste tires (e.g., sand, rocks) before their grinding. The sulfur content in modified GTR was in the range of 0.35–1.17%. It is an essential element as it forms cross-linking bonds in rubber structure during vulcanization. Although its content affects the cure rate and cross-link density of the obtained materials, it is not possible to determine it based on the obtained results. Similar to the study [61], no correlation between the sulfur content in samples and their cross-link density was observed. It might be related to the structure of cross-linking bonds, containing various amounts of sulfur. Calcium, aluminum, and bromine were also detected in small quantities, which are probably impurities in rubber compounds. Trace amounts of iron indicate residues from the steel wires removed when dismantling a waste tires for recycling.

### 3.5. Volatile Organic Compound Emission Profile Determined for Modified GTR

**Figure 5:**
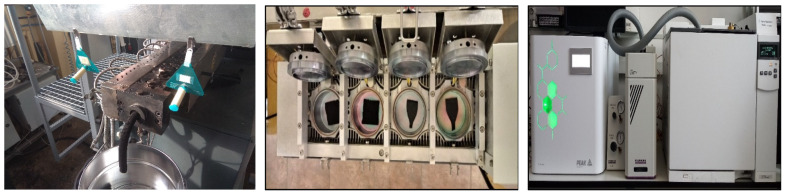
The appearance of the apparatus for determining the TVOCs parameter; from left: Radiello^®^ passive sampling system; Micro-Chamber/Thermal Extractor^TM^; gas chromatograph.

Two types of sampling were used to determine the emission of volatile organic compounds. Firstly, the compounds collected by the Radiello^®^ passive sampling device during reactive extrusion and curing of modified GTR were analyzed. Secondly, prepared materials (uncured and cured) were tested during heating in special chambers by two techniques under different conditions. The VOCs were analyzed by both gas chromatography with mass spectrometry (GC-MS) and gas chromatography with a flame ionization detector (GC-FID). The apparatus used to carry out the above analyzes is shown in Figure 5.

The type of chromatography used allows for different forms of results. GC-MS allows for identifying chemical structures and their concentration, while GC-FID provides information about total volatile organic compounds (TVOCs) emitted from the surface of the tested materials.

Based on the collection via Radiello^®^ passive sampling unit during reactive extrusion and curing, it was found that the determined VOCs released from modified GTR during these processes practically coincide with each other. The chemical structures of identified compounds are presented in Table 7. It can be seen that the determined compounds are mostly degradation products of GTR, and more specifically, natural rubber and styrene-butadiene rubber by-products. The highest concentration among VOCs was noticed for toluene, styrene, benzaldehyde, and limonene, which indicates that the primary reclaiming mechanism during extrusion is the scission of the rubber main chains. It should be mentioned that the presence of these compounds in the VOCs identified during RPA analysis proves the occurrence of the reclaiming process (chains scission) also during crosslinking.

Table 8 presents TVOCs determined by the Radiello^®^ passive sampling unit during reactive extrusion and by Micro-Chamber/Thermal Extractor^TM^ and SHS-GC-MS analysis after extrusion (uncured samples) and after curing of modified GTR.

The TVOCs measured during 30 min reactive extrusion process of proposed materials were in the range of 6.4–10.1 µg. The type and content of modifier did not significantly affect the emission value obtained. It is worth mentioning that the efficiency of the extrusion process is above 3 kg/h; therefore, the TVOCs detected seem to be very low. It should be highlighted that the developed method of producing modified reclaimed rubber is not only low-temperature but also low-emission, which makes the process even more environmentally friendly.

The values of TVOCs parameter determined using Markes’ Micro-Chamber/Thermal Extractor^TM^ and TD-GC-FID system were in the range of 2.8–5.9 µg/g and 44.8–75.3 µg/g, whereas the same parameter obtained by SHS-GC-MS measurement was on average 82 µg/g and 1296 µg/g for uncured and cured samples, respectively. Considering the data listed in Table 8, clear differences between obtained results of TVOCs parameter with the use of two different analytical techniques might be observed. Nevertheless, the main trend associated with the differences in the emissions of VOCs from investigated GTR samples (depending on their composition) was similar. This phenomenon might be the reason to state that both techniques might be employed to evaluate the quality of prepared modified GTR samples with a different composition. Taking into account the characteristics of the operating conditions of the applied analytical devices and considering parameters of the used analytical procedures, the differences between the obtained TVOCs results are influenced by such aspects as: (i) devices operating/sampling mode—SHS-GC-MS system works in a static/equilibrium sampling mode, while the µ-CTE™ 250 works in an active/dynamic non-equilibrium sampling mode; (ii) analytes sampling technique—using µ-CTE™ 250 the VOCs emitted to the gas phase were collected on the sorption medium (Tenax TA) on which the analytes were isolated and enriched, while the static headspace analysis collect and inject only the defined small volume of a gas phase sample (after sample equilibrating stage); (iii) GC equipment (mainly differences in the applied capillary column); (iv) samples injection systems—in a case of SHS-GC-MS system the introduction of a gas sample into the GC column was performed automatically with the use of injection loop (clearly defined internal volume) while using µ-CTE™ 250 and Tenax TA tubes analytes injection adsorbed on a sorption medium was performed with the use of thermal desorption unit (gives a possibility to narrow the chromatographic window); (v) internal volume and loading factor of applied chambers—each of stainless steel chamber has internal volume of 114 cm^3^ while the volume of single glass vial for headspace analysis is 22 mL. For this reason, the loading factor in the case of headspace glass vials is higher than for stainless steel emission chamber; (vi) mass of investigated samples; (vii) calibration protocols and working parameters of applied GC detectors.

Regardless of the technique used, it was estimated that the total amount of VOCs emitted from the cured modified GTR was about 10–20 times higher in comparison to uncured materials. This is related to the higher pressing temperature, which favors the degradation of the material. This, in turn, results in the release of decomposition by-products into the atmosphere during subsequent heating. It is worth mentioning that the TVOCs determined by Wiśniewska et al. [67] using the same Micro-Chamber/Thermal Extractor^TM^ system for uncured modified GTR is around 17.8–29.2 µg/g while the processing temperature was set at 130 °C. These values are higher than those achieved in this work (2.8–5.9 µg/g) for modified reclaimed rubber processed at 60 °C. This allows the conclusion that there is a correlation between the processing temperature and the emission level. It seems that in the case of GTR-based materials, the more important parameter is the temperature of material after extrusion and its cooling method/conditions (if applied), which will affect the volatile organic compounds emission profile.

**Figure 6:**
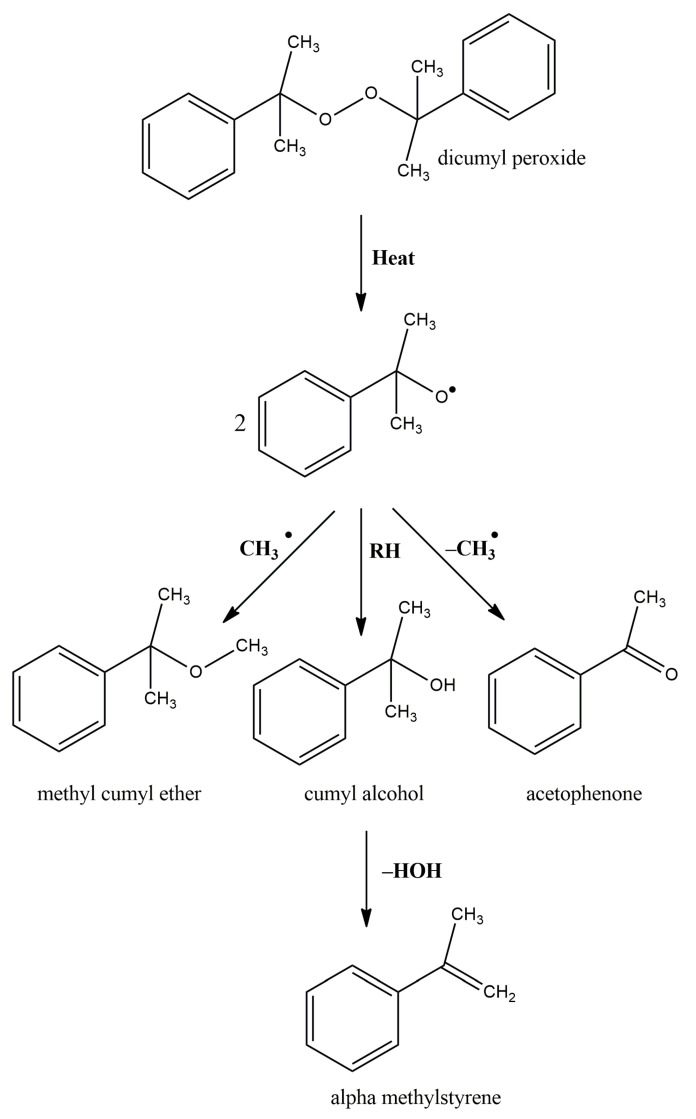
Mechanism of dicumyl peroxide decomposition.

Table 9 and Table 10 present the concentration of the most significant VOCs determined for uncured and cured samples using Micro-Chamber/Thermal Extractor^TM^ system and SHS-GC-MS analysis, respectively. Identified compounds such as α-methyl styrene, acetophenone, α-cumyl alcohol, and methyl cumyl ether are decomposition by-products of dicumyl peroxide [64,68,69,70]. The mechanism of dicumyl peroxide decomposition is shown in Figure 6. As expected, the intensity of released DCP decomposition by-products in cured samples is much higher than for uncured modified GTR according to both techniques. This is due to the high pressing temperature (170 °C), which caused partial decomposition of dicumyl peroxide. According to DSC experimental data investigated by Lv et al. [71], DCP initial decomposition temperature was 133–143 °C. Interestingly, in the cured samples, the decomposition products of DCP make up as much as 96–97% of the TVOCs. Additionally, it is worth mentioning that one of the identified compounds (by Micro-Chamber/Thermal Extractor^TM^) is benzothiazole which is a vulcanization accelerator residue. This substance in VOCs proves the disintegration of cross-linking bonds in reclaimed rubber during reactive extrusion and should be considered an “indicator” or “marker” of devulcanization progress. The reduction of the benzothiazole concentration in the cured materials results from effective cross-linking during compression molding.

## 4. Conclusions

In this paper, GTR was modified by low-temperature extrusion in the presence of a low amount of commercially available elastomeric modifiers. The reclaiming process was analyzed regarding energy consumption and volatile organic compounds emission. Additionally, the mechanical properties and swelling behavior of the obtained samples were investigated.

The results show that processing of GTR with 15 phr of modifier requires slightly more energy compared to GTR modified by 2.5 phr of elastomer while the energy consumption related to heating barrels was 50–100% lower, thanks to the self-heating phenomenon of the materials. The total amount of VOCs measured during the 30 min reactive extrusion process (with efficiency above 3 kg/h) of the proposed materials was in the range of 6.4–10.1 µg. The VOCs analysis for cured samples showed that above 90% of emitted gases are decomposition by-products of dicumyl peroxide, while the level of benzothiazole (devulcanization “marker”) was very low.

Mechanical properties of GTR modified by SBR or SEBS-*g*-MA were much better compared to EOC. A small addition of the modifier improved the tensile strength from 5.2 to 8.1 MPa and from 5.0 to 6.9 MPa and elongation at break from 113 to 136% and from 120 to 170% for GTR_SBR_ and GTR_SEBS-*g*-MA_, respectively.

An important aspect discussed in this study is the huge dependence of VOCs measurements results on the methodology used. Therefore, it seems that in the near future the appropriate methodology and normalization standards should be defined for this purpose. This approach should also consider more complex characteristics (e.g., composition, degradation degree, etc.) of waste rubber, which is currently usually limited only to average particle size or particle size distribution. Otherwise, the true value of the VOCs emission level would be easy to manipulate (by appropriate selection of recycled rubber source or test conditions). Moreover, further investigations in that field of research should focus on optimization and up-scaling of GTR modification via low-temperature extrusion, which can be achieved by suitable shear forces generated by specially designed screw configuration, high-speed mixers, or multi-screw extruders. Another interesting direction for future development is finding new additives/modifiers (e.g., from renewable resources or waste materials) dedicated to improving processing or physico-mechanical properties.

## Figures and Tables

**Table 1:** Sample coding, GTR modification and formulation procedure.

Sample Coding	GTR_XY_	X—Modifier Type: EOC; SBR or SEBS-*g*-MA	Y—The Amount of Modifier: 2.5; 5; 10 and 15 phr
**GTR Modification**	Modification was performed using a co-rotating twin screw extruder EHP 2 × 20 Sline with an L/d ratio of 40 and d = 20 mm produced by Zamak Mercator (Skawina, Poland). Rotational screw speed: 150 rpm Barrel temperature (from hopper to extrusion die): 35/60/60/60/60/60/60/25/25/25 °C Prior to extrusion, a premix of GTR and DCP (2 phr) was prepared. GTR/DCP premix and elastomeric modifier were dosed with a total throughput: 3 kg/h.		
**Modified GTR Formulation**	Modified GTR samples were formed into sheets of about 2 mm using hydraulic press PH-90 manufactured by ZUP Nysa (Nysa, Poland) Temperature: 170 °C, Pressure: 9.8 MPa Samples were compressed according to the optimal vulcanization time determined by ISO 6502 standard.		

**Table 2:** The temperature at die and extruder energy consumption measured during GTR modification.

Sample Code	Temperature at Die (°C)	SME (kWh/kg)	Extruder Energy Consumption (kWh/kg)
GTR_SBR2.5_	46 ± 1	0.131 ± 0.002	0.433 ± 0.030
GTR_SBR5.0_	50 ± 2	0.136 ± 0.003	0.440 ± 0.025
GTR_SBR10.0_	92 ± 4	0.250 ± 0.007	0.580 ± 0.022
GTR_SBR15.0_	96 ± 1	0.262 ± 0.004	0.587 ± 0.021
GTR_SEBS-*g*-MA2.5_	43 ± 1	0.090 ± 0.004	0.407 ± 0.039
GTR_SEBS-*g*-MA5.0_	42 ± 1	0.111 ± 0.007	0.413 ± 0.021
GTR_SEBS-*g*-MA10.0_	44 ± 1	0.123 ± 0.008	0.433 ± 0.030
GTR_SEBS-*g*-MA15.0_	47 ± 2	0.145 ± 0.006	0.440 ± 0.044
GTR_EOC2.5_	44 ± 1	0.091 ± 0.005	0.420 ± 0.022
GTR_EOC5.0_	78 ± 2	0.138 ± 0.011	0.453 ± 0.021
GTR_EOC10.0_	79 ± 2	0.176 ± 0.006	0.480 ± 0.025
GTR_EOC15.0_	81 ± 1	0.195 ± 0.002	0.487 ± 0.030

**Table 3:** Curing characteristics of modified GTR determined at 170 °C.

Sample Code	Curing Parameters						
	M_L_ (dNm)	M_H_ (dNm)	ΔM (dNm)	t_2_ (min.)	t_90_ (min.)	CRI (min^−1^)	R_300_ (%)
GTR_SBR2.5_	10.8	19.9	9.1	0.4	5.6	19.0	0.6
GTR_SBR5.0_	10.2	18.2	8.0	0.5	5.7	19.4	0.5
GTR_SBR10.0_	7.2	14.9	7.7	0.2	5.3	19.8	0.6
GTR_SBR15.0_	6.5	13.1	6.6	0.2	5.4	19.3	0.7
GTR_SEBS-*g*-MA2.5_	10.9	20.3	9.4	0.4	5.7	18.8	0.4
GTR_SEBS-*g*-MA5.0_	8.8	19.4	10.6	0.2	5.6	18.7	0.2
GTR_SEBS-*g*-MA10.0_	9.9	16.4	6.6	0.6	6.1	18.0	0.3
GTR_SEBS-*g*-MA15.0_	9.3	15.4	6.2	0.6	6.3	17.5	0.3
GTR_EOC2.5_	9.6	19.9	10.3	0.1	5.4	19.0	0.6
GTR_EOC5.0_	8.3	16.9	8.7	0.3	5.1	21.0	0.9
GTR_EOC10.0_	6.4	14.8	8.4	0.3	5.4	19.6	0.6
GTR_EOC15.0_	5.2	12.7	7.4	0.4	6.2	17.2	0.4

**Table 4:** Physico-mechanical properties of modified GTR.

Sample Code	Tensile Strength (MPa)	Elongation at Break (%)	Hardness (Shore A)	Density (g/cm^3^)	Swelling Degree (%)	Sol Fraction (%)	Cross-Link Density (mol/cm^3^ × 10^−4^)
GTR_SBR2.5_	5.2 ± 0.3	113 ± 8	71 ± 1	1.162 ± 0.002	122 ± 4	9.4 ± 0.2	1.30 ± 0.07
GTR_SBR5.0_	6.4 ± 0.3	127 ± 7	75 ± 1	1.158 ± 0.005	133 ± 1	9.1 ± 0.1	1.13 ± 0.01
GTR_SBR10.0_	7.5 ± 0.1	133 ± 4	81 ± 1	1.149 ± 0.002	146 ± 1	7.9 ± 0.1	1.01 ± 0.02
GTR_SBR15.0_	8.1 ± 0.3	136 ± 4	84 ± 1	1.144 ± 0.001	157 ± 1	7.0 ± 0.2	0.92 ± 0.01
GTR_SEBS-*g*-MA2.5_	5.0 ± 0.1	120 ± 6	66 ± 1	1.155 ± 0.001	125 ± 3	9.5 ± 0.1	1.25 ± 0.04
GTR_SEBS-*g*-MA5.0_	5.6 ± 0.2	134 ± 3	66 ± 1	1.144 ± 0.004	138 ± 0	9.4 ± 0.1	1.07 ± 0.01
GTR_SEBS-*g*-MA10.0_	6.3 ± 0.1	148 ± 6	67 ± 1	1.126 ± 0.001	151 ± 2	9.3 ± 0.2	0.94 ± 0.03
GTR_SEBS-*g*-MA15.0_	6.9 ± 0.1	170 ± 5	67 ± 1	1.112 ± 0.002	172 ± 1	9.2 ± 0.1	0.77 ± 0.01
GTR_EOC2.5_	4.5 ± 0.1	126 ± 3	64 ± 1	1.150 ± 0.001	131 ± 1	10.0 ± 0.2	1.13 ± 0.02
GTR_EOC5.0_	4.1 ± 0.1	130 ± 4	63 ± 1	1.138 ± 0.003	141 ± 1	9.7 ± 0.2	1.03 ± 0.02
GTR_EOC10.0_	3.8 ± 0.2	139 ± 5	61 ± 1	1.121 ± 0.001	167 ± 4	10.5 ± 0.1	0.76 ± 0.03
GTR_EOC15.0_	3.5 ± 0.3	143 ± 8	60 ± 1	1.100 ± 0.001	187 ± 3	10.4 ± 0.3	0.64 ± 0.02

**Table 5:** Comparison of tensile properties of GTR/SBR blends described in the literature.

Sample Composition	Sample Preparation	Tensile Strength (MPa)	Elongation at Break (%)	Hardness (Sh A)	References
GTR/SBR + DCP 100/2.5, 100/5, 100/10, 100/15	Extrusion at 60 °C; compression molding at 170 °C	5.2 ± 0.3 6.4 ± 0.3 7.5 ± 0.1 8.1 ± 0.3	113 ± 8 127 ± 7 133 ± 4 136 ± 4	71 ± 1 75 ± 1 81 ± 1 84 ± 1	This study
LDPE/SBR/GTR + DCP 50/25/25	Two-roll mills at 60 °C (GTR and SBR); internal mixer at 130 °C at a rotor speed of 60 rpm (LDPE, GTR/SBR, and DCP); compression molding at 135 °C	4.1	33	82	[56]
SBR/GTR + sulfur system 50/50	Microwave devulcanization of GTR; two-roll mill at room temperature; compression molding at 170 °C	4.7–4.9	366–445	66–67	[57]
SBR/GTR + sulfur system 40/60	Two-roll mills at room temperature; compression molding at 160 °C	5.0	445	60	[58]
SBR/GTR + sulfur system 0/100, 10/90, 20/80	Mechano-chemical devulcanization of GTR; two-roll mills at 50 °C; compression molding at 142 °C	3.1 4.8 6.0	100 160 200	-	[59]
PP/SBR/GTR + DCP 30/40/30	Internal mixer at 185 °C at a rotor speed of 60 rpm; injection molding at 240 °C	10–11	175–225	-	[60]

**Table 6:** XFR measurement results for studied materials.

		Element (wt.%)						
		Zn	Si	S	Ca	Al	Br	Fe
Uncured	GTR_SBR2.5_	0.88	0.71	0.82	0.19	0.05	0.03	0.02
	GTR_SBR15.0_	0.56	0.40	0.40	0.12	0.05	0.02	0.01
	GTR_SEBS-*g*-MA2.5_	1.17	0.87	0.82	0.22	-	0.04	0.03
	GTR_SEBS-*g*-MA15.0_	0.91	0.67	0.65	0.21	0.04	0.03	0.02
	GTR_EOC2.5_	1.01	0.76	0.72	0.22	0.05	0.04	0.02
	GTR_EOC15.0_	0.68	0.42	0.35	0.14	0.07	0.02	0.02
Cured	GTR_SBR2.5_	1.10	1.18	1.04	0.25	0.11	0.04	0.03
	GTR_SBR15.0_	1.01	0.97	0.91	0.22	0.15	0.04	0.02
	GTR_SEBS-*g*-MA2.5_	0.96	1.09	0.99	0.20	0.08	0.03	0.02
	GTR_SEBS-*g*-MA15.0_	1.07	1.15	0.89	0.24	0.08	0.04	0.02
	GTR_EOC2.5_	1.27	1.28	1.17	0.29	0.12	0.05	0.03
	GTR_EOC15.0_	1.02	0.92	0.83	0.23	0.11	0.04	0.02

**Table 7:** Volatile organic compounds emitted during curing of modified GTR determined by GC-MS analysis.

Retention Time (min)	Identified Compound	Chemical Structure	Molecular Weight (g/mol)	Match Quality (%)	Source	References
4.02	benzene	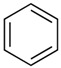	78.11	91	styrene-butadiene rubber present in GTR	[62]
5.30	toluene	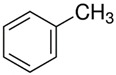	92.14	94	styrene-butadiene rubber present in GTR	[62,63]
6.73	xylene	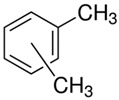	106.17	97	styrene-butadiene rubber present in GTR	[62,63]
7.04	styrene	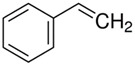	104.15	97	styrene-butadiene rubber present in GTR	[62,63]
8.12	benzaldehyde	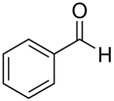	106.12	96	styrene-butadiene rubber present in GTR	[62]
8.78	α-methylstyrene	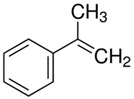	118.18	96	dicumyl peroxide decomposition	[64]
9.68	cymene	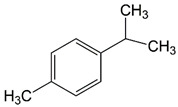	134.22	94	styrene-butadiene rubber present in GTR	
9.89	limonene	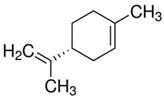	136.23	94	natural rubber present in GTR	[28,65,66]
10.18	acetophenone	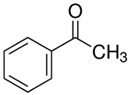	120.15	94	dicumyl peroxide decomposition	[64]
12.93	dodecene	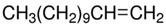	168.32	96	aliphatic thermplastics and natural rubber present in GTR	-
16.01	tetradecane	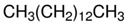	198.39	98	aliphatic thermplastics and natural rubber present in GTR	-

**Table 8:** TVOCs parameter measured by different techniques at a different time of preparing modified GTR samples.

Sample Code	TVOCs [µg] Measured	TVOCs [µg/g] Measured			
	During Extrusion (Radiello^®^)	After Extrusion (Micro-Chamber/ Thermal Extractor^TM^)	After Extrusion (SHS-GC-MS)	After curing (Micro-Chamber/ Thermal Extractor^TM^)	After Curing (SHS-GC-MS)
GTR_SBR2.5_	9.8	4.5	82	44.8	1645
GTR_SBR15.0_	10.1	5.7	130	48.9	1965
GTR_SEBS-*g*-MA2.5_	-	3.3	79	75.3	1608
GTR_SEBS-*g*-MA15.0_	7.2	5.9	80	67.5	316
GTR_EOC2.5_	9.3	3.0	71	60.8	1854
GTR_EOC15.0_	6.4	2.8	48	57.1	227

**Table 9:** Concentration of the most significant VOCs identified for uncured and cured materials by micro-chamber/thermal extractor system.

	Identified VOC	α-Methylstyrene		Acetophenone		α-Cumyl Alcohol		Methyl Cumyl Ether		Benzothiazole	
	Concentration	(µg/g)	(% TVOC)	(µg/g)	(% TVOC)	(µg/g)	(% TVOC)	(µg/g)	(% TVOC)	(µg/g)	(% TVOC)
Uncured	GTR_SBR2.5_	0.2	5.2	0.4	8.6	0.9	20.0	-	-	0.3	5.8
	GTR_SBR15.0_	0.2	4.2	0.7	11.7	3.0	52.1	<0.1	0.3	0.1	2.2
	GTR_SEBS-*g*-MA2.5_	0.1	3.0	0.2	6.4	0.4	11.2	-	-	0.3	8.0
	GTR_SEBS-*g*-MA15.0_	0.2	3.9	0.5	9.1	2.1	35.7	<0.1	0.1	0.2	4.2
	GTR_EOC2.5_	0.1	1.8	0.1	4.3	0.4	13.4	-	-	0.3	8.3
	GTR_EOC15.0_	0.1	1.5	0.1	3.0	0.3	11.8	<0.1	0.2	0.2	8.4
Cured	GTR_SBR2.5_	1.1	2.6	5.4	12.2	35.0	78.3	1.4	3.2	0.1	0.2
	GTR_SBR15.0_	1.5	3.0	9.1	18.5	34.8	71.2	2.0	4.1	0.1	0.3
	GTR_SEBS-*g*-MA2.5_	2.1	2.8	9.6	12.7	59.3	78.8	2.0	2.6	0.2	0.2
	GTR_SEBS-*g*-MA15.0_	1.9	2.8	11.5	17.0	49.5	73.3	2.2	3.3	0.1	0.2
	GTR_EOC2.5_	1.6	2.6	7.8	12.9	47.7	78.5	1.6	2.7	0.2	0.3
	GTR_EOC15.0_	1.7	3.0	8.7	15.3	43.4	76.0	1.6	2.8	0.2	0.3

**Table 10:** Concentration of the most significant VOCs identified for uncured and cured materials by SHS-GC-MS analysis.

		Concentration (µg/g)				
		α-Methylstyrene	Acetophenone	α-Cumyl Alcohol	Methyl Cumyl Ether	Benzothiazole
Uncured	GTR_SBR2.5_	12	7	26	2	-
	GTR_SBR15.0_	36	14	34	3	-
	GTR_SEBS-*g*-MA2.5_	9	6	15	-	-
	GTR_SEBS-*g*-MA15.0_	9	-	29	3	-
	GTR_EOC2.5_	4	10	17	-	-
	GTR_EOC15.0_	-	-	-	-	-
Cured	GTR_SBR2.5_	7	89	1515	12	3
	GTR_SBR15.0_	8	251	1656	24	5
	GTR_SEBS-*g*-MA2.5_	6	71	1498	13	-
	GTR_SEBS-*g*-MA15.0_	7	32	243	10	-
	GTR_EOC2.5_	4	163	1659	15	4
	GTR_EOC15.0_	20	24	146	12	-

## Data Availability

Not applicable.

## Supplementary Materials

The following supporting information can be downloaded at: https://www.mdpi.com/article/10.3390/polym14030546/s1, Table S1: Characteristic of sampling protocol applied to collect the VOCs emitted to the gas phase/indoors during reactive extrusion and curing characteristics by rubber process analyzer (RPA). Table S2: Thermal desorption (TD) GC-FID and GC-MS system working parameters used to assess the type and amount of VOCs emitted to the gas phase/indoors during reactive extrusion, as well as in the case of emissions of VOCs from prepared modified GTR samples. Table S3: General description of sampling/conditioning protocol used to estimate the emissions of VOCs released from the surface of prepared modified GTR samples.
