# Supplementary material for: Ground Tire Rubber Modified by Elastomers via Low-Temperature Extrusion Process: Physico-Mechanical Properties and Volatile Organic Emission Assessment
Source: Polymers (Basel). 2022 Jan 28;14(3):546. doi: 10.3390/polym14030546 (PMC8839703; doi:10.3390/polym14030546)
Supplement: Supplementary file 1 [file polymers-14-00546-s001.zip › polymers-1544194-supplementary.pdf]

Table S1. Characteristic of sampling protocol applied to collect the VOCs emitted to the gas phase/indoors during reactive extrusion and curing characteristics by rubber process analyzer (RPA).

Sampling of VOCs directly from the extrusion die was performed for 30 minutes while in case of RPA for 20 minutes (duration of the measurement) using Radiello® diffusive passive sampler	
Radiello® diffusive passive sampler characteristic	
Diffusion membrane made of sintered polyethylene	Length, external diameter and thickness – 60 mm x 16 mm x 5 mm;<br>Diffusion zone length – 150 mm
Cylindrical cartridge made of stainless steel net filled with graphitised charcoal Carbograph 4	Length and external diameter – 60 mm x 4.8 mm;<br>Sorbent mass – 300 ± 10 mg
After the sampling period, the cylindrical containers were placed in glass tubes, closed with PE nut, and transported to the laboratory	
The liberation process of VOCs collected on the Carbograph 4 was performed using a two-stage thermal desorption technique	

Table S2. Thermal desorption (TD) GC-FID and GC-MS system working parameters used to assess the type and amount of VOCs emitted to the gas phase/indoors during reactive extrusion, as well as in the case of emissions of VOCs from prepared modified GTR samples.

**Working conditions of the two-stage thermal desorption unit**		
Analytical procedure acronym	TD-GC-FID	TD-GC-MS
Applied thermal desorber	Markes' Series 2 Thermal Desorption System; UNITY/TD-100	Unity v.2, Markes International Ltd.
Steel tube heating time and temperature at the 1st stage of thermal desorption	sample/tube temp. – 290 °C;<br>desorption time – 12 min;<br>gas flow rate – 50 mL/min;<br>microtrap temperature – 0 °C;	
Microtrap heating time and temperature at the 2nd stage of thermal desorption	microtrap temp. – 300 °C;<br>ballistic heating time – 5 min	
Flow rate of the inert gas (He) through the microtrap to the chromatographic column	2.0 mL/min	1.0 mL/min
**Working conditions of the final determination system**		
Gas chromatograph	Agilent 7820A GC	Agilent Technologies 6890
Detector	Flame ionisation detector, detector temp. 280 °C	Mass spectrometer (5873 Network Mass Selective Detector, Agilent Technologies);<br>transmission line temp. of GC-MS: 150 °C;<br>ion source temp. – 230 °C;<br>quadrupole mass analyser temp. – 150 °C
Transfer line temperature TD-GC	180 °C	160 °C
Capillary column	DB-1 (30 m × 0.32 mm; stationary phase thickness – 5 µm, J&W, USA);<br>helium flow rate – 2.0 mL/min	HP-1MS (30 m × 0.25 mm; stationary phase thickness – 1 µm, J&W, USA);<br>helium flow rate – 1.0 mL/min
Temperature programme	45 °C for 1 min;<br>15 °C/min to 120 °C hold for 2 min;<br>10 °C/min up to 250 °C hold for 5 min	50 °C for 1 min;<br>15 °C/min up to 120 °C and hold for 2 min;<br>10 °C/min up to 260 °C and hold for 5 min

Table S3. General description of sampling/conditioning protocol used to estimate the emissions of VOCs released from the surface of prepared modified GTR samples**.**

<table>
<tr><td>Average mass of investigated samples</td><td>uncured modified GTR - 1.17 ± 0.24 g;
cured modified GTR - 2.60 ± 0.32 g</td></tr>
<tr><td>Sampling/conditioning device</td><td>miniature emission chambers system μ-CTE™ 250 (Markes' Micro-Chamber/Thermal Ex-tractor™, Markes International, Inc) working in a dynamic analytes sampling mode</td></tr>
<tr><td>Micro-Chamber/Thermal Extractor™ seasoning conditions of prepared rubber samples</td><td>samples conditioning temperature – 40 °C;
sampling time – 40 min;
nitrogen gas flow rate through a single chamber – 11.5 mL/min</td></tr>
<tr><td>Analytes sampling device</td><td>Stainless steel tubes filled with Tenax TA (60/80 mesh, stainless steel tube, Merck KGaA, Darmstadt, Germany)</td></tr>
<tr><td colspan="2">After the sampling, sorption tubes were removed from the outlets of chambers and sealed from both sides with braze nuts</td></tr>
<tr><td colspan="2">The liberation process of VOCs collected on the Tenax TA was performed using a two-stage thermal desorption technique</td></tr>
</table>
